# Differences in acute phase response to bacterial, fungal and viral antigens in greater mouse-eared bats (*Myotis myotis*)

**DOI:** 10.1038/s41598-022-18240-6

**Published:** 2022-09-10

**Authors:** Anne Seltmann, Sara A. Troxell, Julia Schad, Marcus Fritze, Liam D. Bailey, Christian C. Voigt, Gábor Á. Czirják

**Affiliations:** 1 Department of Evolutionary Ecology, Leibniz Institute for Zoo and Wildlife Research, Alfred-Kowalke-Str. 17, 10315 Berlin, Germany; 2 Institute of Biology, Freie Universität Berlin, Takustr. 3, 14195 Berlin, Germany; 3 Department of Evolutionary Genetics, Leibniz Institute for Zoo and Wildlife Research, Alfred-Kowalke-Str. 17, 10315 Berlin, Germany; 4 Department of Wildlife Diseases, Leibniz Institute for Zoo and Wildlife Research, Alfred-Kowalke-Str. 17, 10315 Berlin, Germany; 5 Present Address: Department 3: Public Relations and Environmental Education, National Park Administration Saxon Switzerland, An der Elbe 4, 01814 Bad Schandau, Germany; 6 Present Address: Applied Zoology and Nature Conservation, University of Greifswald, Loitzer Str. 26, 17489 Greifswald, Germany

**Keywords:** Innate immunity, Ecophysiology

## Abstract

The acute phase response (APR) is an evolutionarily well-conserved part of the innate immune defense against pathogens. However, recent studies in bats yielded surprisingly diverse results compared to previous APR studies on both vertebrate and invertebrate species. This is especially interesting due to the known role of bats as reservoirs for viruses and other intracellular pathogens, while being susceptible to extracellular microorganisms such as some bacteria and fungi. To better understand these discrepancies and the reservoir-competence of bats, we mimicked bacterial, viral and fungal infections in greater mouse-eared bats (*Myotis myotis*) and quantified different aspects of the APR over a two-day period. Individuals reacted most strongly to a viral (PolyI:C) and a bacterial (LPS) antigen, reflected by an increase of haptoglobin levels (LPS) and an increase of the neutrophil-to-lymphocyte-ratio (PolyI:C and LPS). We did not detect fever, leukocytosis, body mass loss, or a change in the overall functioning of the innate immunity upon challenge with any antigen. We add evidence that bats respond selectively with APR to specific pathogens and that the activation of different parts of the immune system is species-specific.

## Introduction

Bats are an important mammalian order with respect to their biological diversity, but also to their epidemiological role, especially as putative reservoirs for several viral zoonotic pathogens^1^. Interestingly, bats tolerate many viral infections without showing clinical symptoms due to co-evolutionary adaptations^1,2^. In addition to viruses, bats harbour other zoonotic, especially intracellular, pathogens^3^: bacteria, e.g. *Bartonella* spp.^4–6^, fungi, e.g. *Histoplasma capsulatum*^7^ or protozoa, e.g. *Plasmodium* related parasites^8^. However, besides their role as reservoirs, they are vulnerable to various extracellular pathogens and parasites, showing pathological lesions during different bacterial^9–11^ and fungal^1,12–14^ infections. One of these pathogens, the fungus *Pseudogymnoascus destructans*, causes white-nose syndrome in North America resulting in mass mortalities in cave-dwelling hibernating bats with important economic and conservation consequences^15,16^. Over the past years, several immunogenetic and molecular immunological studies addressed why bats may be extraordinarily potent reservoirs for viruses^17,18^. However, our knowledge on their functional immune defenses against other pathogens, especially at the organismic level, is still limited^3^.

Bats have an extraordinary longevity^19^ and long-lived animals are considered to invest more in the adaptive part than in the innate part of the immune system^20^. However, the innate immune system is the first line of defense against pathogens and parasites, bats may fight pathogens already at an early infection state as have been shown with the interferon mediated anti-viral responses both in vitro and in vivo^17^. Once pathogens pass through the constitutive part of the innate immunity (e.g. antibacterial proteins and complement system), they are encountered by various phagocytic cells which recognize them via specific pathogen recognition systems, such as Toll-Like Receptors^21^. Besides aiming to kill the intruders, these cells, especially macrophages and dendritic cells, also initiate an acute phase response (APR)^22,23^. The APR is triggered by a signal cascade beginning with phagocytic cells that release pro-inflammatory cytokines^21^. An APR includes (1) leukocytosis, i.e. the increase in white blood cells, especially neutrophils, as a result of bone marrow activation; (2) fever and (3) sickness behavior, e.g. lethargy, anorexia^24,25^ and a consequent decrease in body mass^26–29^ which are the effects of the pro-inflammatory cytokines on the hypothalamus, fat tissues and muscles, respectively. Moreover, stimulating the hepatocytes, the liver increases the (4) synthesis of acute phase proteins (e.g. haptoglobin, serum amyloid A, C-reactive protein), which does not only decrease the multiplication of the pathogens, but also act as opsonins. Optimal responses to pathogens are evolutionary adaptive because they increase the chances for survival of the respective individual taking into account internal and external conditions. For example, by rising body temperature (fever) the organism impairs the replication of viruses and bacteria^26,28^ whereas both an overly high or insufficient low body temperature can have detrimental effects on individual fitness and are thus maladaptive. An APR is energetically and pathologically costly^30^ and requires the reallocation of resources from digestion, reproduction and locomotion to immune defense^24,31^. An APR can have severe consequences for the fitness of an individual, e.g. tissue damage, reduced growth, less investment in breeding and feeding activities^22,24,32–35^. Thus, a maximal APR to all pathogens may be maladaptive and only serve as an emergency strategy^36^.

The APR, especially during stimulated bacterial infections via lipopolysaccharide (LPS) challenges, has been intensively studied under laboratory conditions in rodents living in an atypical, low-pathogen risk environment^37–39^, and in captive^40,41^, as well as free-ranging birds^34–36^; yet, studies in wild mammals are scarce. While phytohaemagglutinin and LPS are the most common antigens used to activate the innate immune system in wild birds, in the few studies on wild mammals the antigens applied are more diverse^29,42–46^. A deeper knowledge about the APR in wild mammals, especially bats, may be beneficial for identifying species of conservation interest^47^ and better forecast reservoir species and prioritize surveillance targets^48^, and for the development of treatments against the pathogens they carry. Moreover, simulating different types of infections might reveal further answers why the Chiropteran immune system is adapted to tolerate certain pathogens while remaining vigilant against others^3^.

To further shed light on the extent and specific dynamics of APR in wild animals, specifically bats, we conducted an immune challenge experiment designed to elicit an immune reaction in greater mouse-eared bats (*Myotis myotis*), an abundant, insectivorous bat species widely distributed across Europe. The objective of this study was to test whether the APR varies with antigen identity, as would be expected to optimize the trade-off between an investment in the immune system and other fitness-related traits^31^. Based on pathological and clinical differences observed between different types of pathogens (extracellular vs. intracellular^3^), we predicted that bats respond with an inflammatory response to bacterial and fungal, but not to viral infections. Our prediction on lack of APR to a viral antigen was based on previous studies showing that bats have a constitutive expression of the interferon type I (IFN α and β) with antiviral activity^18^ and also a unique anti-inflammatory response^49^.

## Results

We obtained data on immune traits of 38 individual greater mouse-eared bats injected with either LPS (an endotoxin of gram-negative bacteria cell walls), polyinosinic:polycytidylic acid (PolyI:C, a synthetic double-stranded RNA), zymosan (a fungal glucan) or saline solution (control) for almost all measurements. In one case, blood plasma volume was insufficient to conduct bacterial killing activity (BKA) measurement, and in one case the quality of blood smears was inadequate to conduct total and/or differential leukocyte counts.

### Skin temperature, as a proxy for body temperature

**Figure 1:**
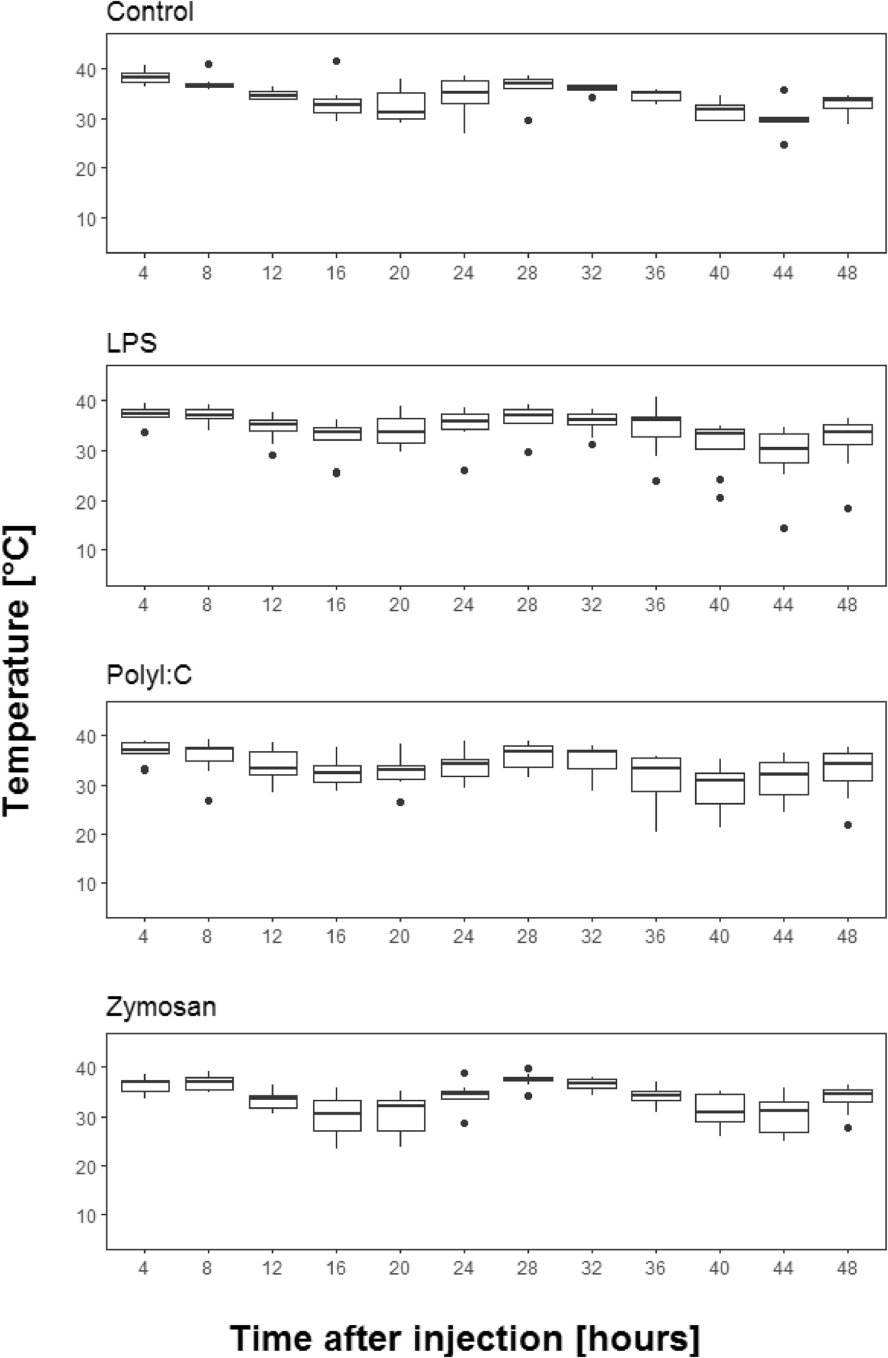
Skin temperature (°C) of bats following injection of saline solution (control), LPS, PolyI:C and zymosan (time after injection = 0 h, not depicted). The figures show medians (bold line), 25–75% percentiles (box), maxima and minima (whiskers) and outliers (dots).

Skin temperature (°C) showed diurnal fluctuations (ANOVA, F = 28.96, df = 11, *p* < 0.001; Fig. 1). We did not find an effect of treatment on skin temperature of bats (F = 0.42, df = 3, *p* = 0.741). The interaction between treatment and time elapsed since injection was also not significant (F = 1.16, df = 33, *p* = 0.261).

### Body mass changes

**Figure 2:**
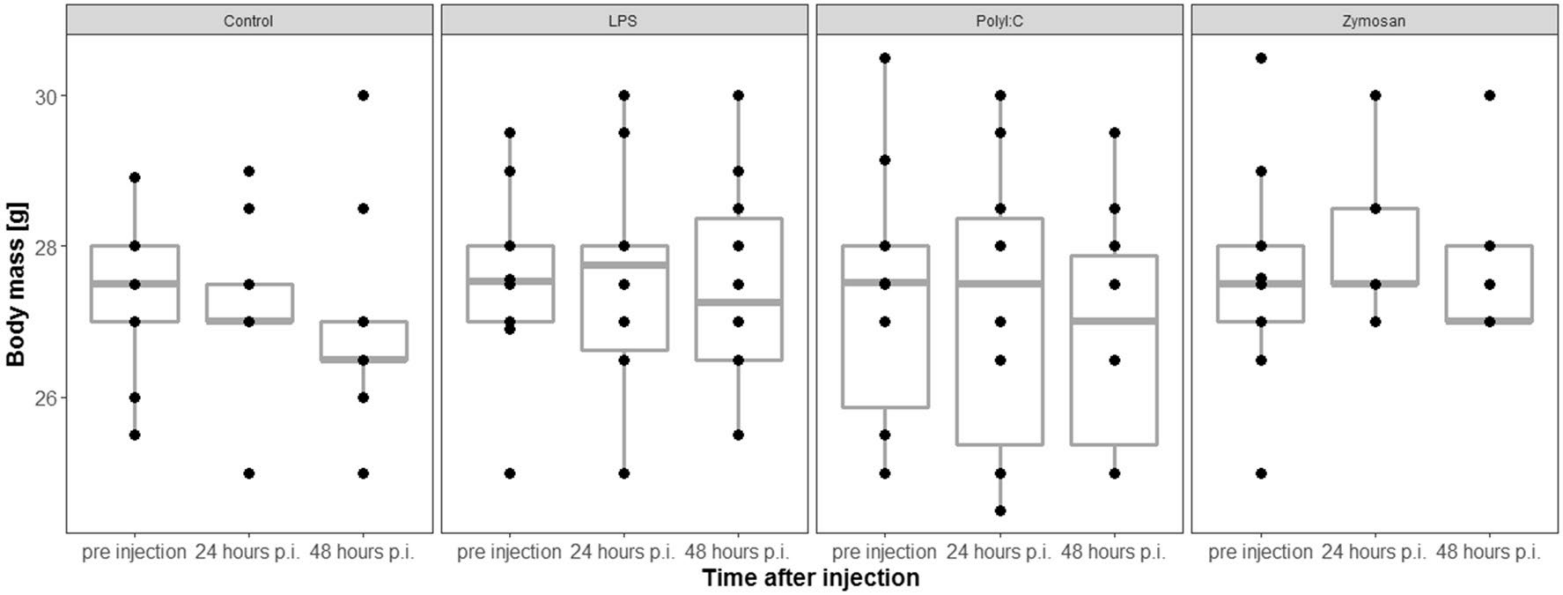
Body mass change was not affected by antigen type. p.i. = post injection. The figures show medians (bold line), 25–75% percentiles (box), maxima and minima (whiskers) and data points (dots).

Pre-injection body mass (g) did not differ across treatment groups (likelihood ratio test (LRT), df = 3, χ^2^ = 0.66, *p* = 0.88, n = 38). Treatment and the interaction between sampling time and treatment did not affect the change in body mass of individuals (LRTs, model with interaction vs. model without interaction: df = 3, χ^2^ = 0.11, *p* = 0.99; model with treatment vs. model without treatment: df = 3, χ^2^ = 4.39, *p* = 0.22, Fig. 2).

### Bacterial killing activity (BKA) changes

Pre-injection BKA (number of viable bacteria after incubation) did not differ across treatment groups (LRT, df = 3, χ^2^ = 6.87, *p* = 0.08, n = 76).

**Figure 3:**
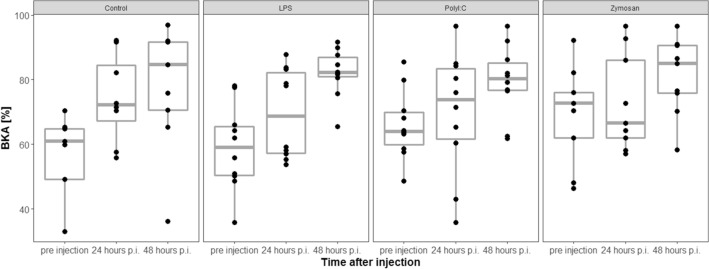
BKA (%) change was not affected by antigen type. p.i. = post injection. The figures show medians (bold line), 25–75% percentiles (box), maxima and minima (whiskers) and data points (dots).

Because the initial number of bacteria was different in the laboratory analyses for samples taken at 24 and 48 h after injection, respectively, we used percentage values as units of measurement as described in the methods section for further statistical analyses. Treatment and the interaction between sampling time and treatment did not affect the change in BKA (%) of individuals (LRTs, model with interaction vs. model without interaction: df = 3, χ^2^ = 2.06, *p* = 0.56; model with treatment vs. model without treatment: df = 3, χ^2^ = 2.3, *p* = 0.51, Fig. 3).

### Haptoglobin level changes

Pre-injection levels of haptoglobin (mg/ml) did not differ across treatment groups (LRT, df = 3, χ^2^ = 0.5, *p* = 0.92, n = 38).

**Figure 4:**
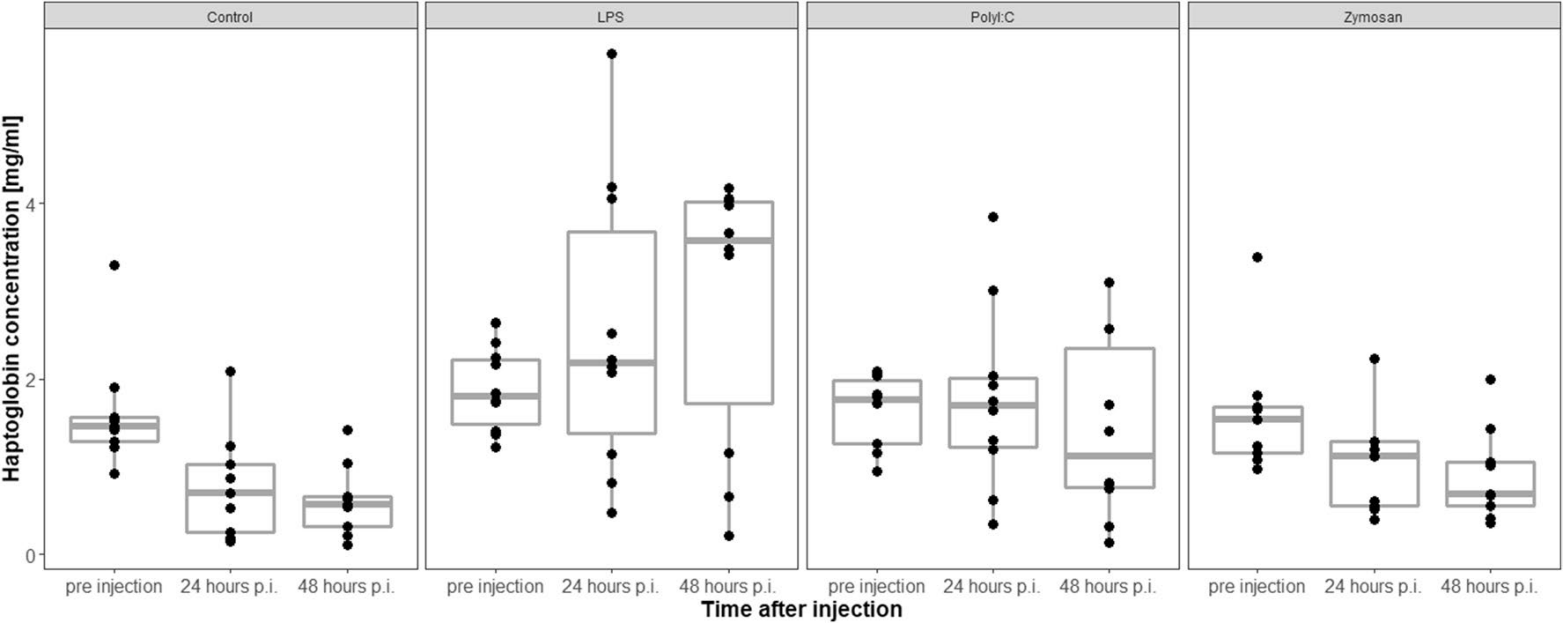
Haptoglobin levels (mg/ml) significantly increased in animals injected with LPS compared to animals injected with saline solution and zymosan. p.i .= post injection. The figures show medians (bold line), 25–75% percentiles (box), maxima and minima (whiskers) and data points (dots).

The interaction between sampling time and antigen did not affect the change in haptoglobin levels of individuals compared to before the injection (LRT, model with interaction between sampling time and treatment vs. model including sampling time and treatment, df = 3, χ^2^ = 2.07, *p* = 0.56, Fig. 4). Treatment affected haptoglobin levels (LRT, model including treatment vs. model without treatment (df = 3, χ^2^ = 13.75, *p* = 0.003). Haptoglobin levels significantly increased with time in animals injected with LPS compared to animals injected with saline solution and zymosan, where haptoglobin levels decreased (general linear hypothesis testing (GLHT), saline solution vs. LPS: Estimate = − 0.38, SE = 0.11, z = − 3.58, *p* = 0.002; zymosan vs. LPS: Estimate = − 0.3, SE = 0.11, z = − 2.81, *p* = 0.025). All other comparisons among treatment groups, including PolyI:C, did not yield significant results (Estimate < 0.22, *p* > 0.17, respectively).

### Total leukocyte count changes

**Figure 5:**
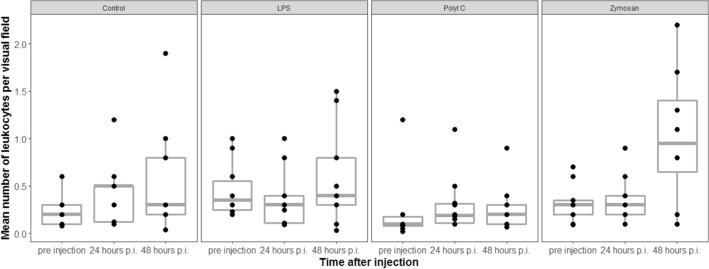
The number of total leukocytes was not affected by antigen type. p.i. = post injection. The figures show medians (bold line), 25–75% percentiles (box), maxima and minima (whiskers) and data points (dots).

The total number of leukocytes did not differ across treatment groups before injection of antigen/saline solution (LRT, df = 3, χ^2^ = 10.15, *p* = 0.92, n = 38). Treatment and the interaction between sampling time and treatment did not affect the change in the total leukocyte counts of individuals compared to before the injection (LRTs, model with interaction vs. model without interaction: df = 3, χ^2^ = 0.78, *p* = 0.85; model with treatment vs. model without treatment: df = 3, χ^2^ = 1.61, *p* = 0.666, Fig. 5).

### Relative numbers of lymphocytes

Relative numbers of lymphocytes (%) did not differ across treatment groups before injection of antigen/saline solution (LRT, df = 3, χ^2^ = 9.64, *p* = 0.92, n = 38).

**Figure 6:**
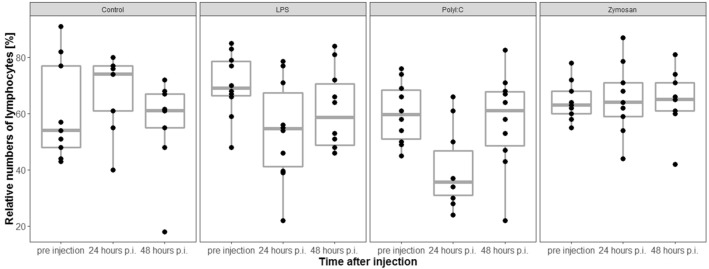
Relative numbers of lymphocytes significantly decreased in LPS- and PolyI:C treated individuals compared with control individuals 24 h p.i. = post injection. The figures show medians (bold line), 25–75% percentiles (box), maxima and minima (whiskers) and data points (dots).

The interaction between treatment and sampling time was significant (LRT, model with interaction vs. model without interaction: df = 3, χ^2^ = 12.36, *p* = 0.006, Fig. 6). Because we were only interested in comparisons across treatments for the same sampling time, we restricted our post hoc analyses to comparisons among treatment at 24 h and 48 h post injection (p.i.), respectively. Relative numbers of lymphocytes decreased in individuals treated with LPS or PolyI:C compared with individuals treated with saline solution 24 h p.i. (GLHT, saline solution vs. LPS: Estimate = − 24.18, SE = 7.52, z = − 3.21; *p* = 0.01; saline solution vs. PolyI:C: Estimate = − 27.88, SE = 7.52, z = − 3.71; *p* = 0.002). There were no differences between other combinations of treatment at 24 or 48 h p.i., respectively (GLHT, Estimate < 20.94; *p* > 0.053, respectively).

### Relative numbers of neutrophils

**Figure 7:**
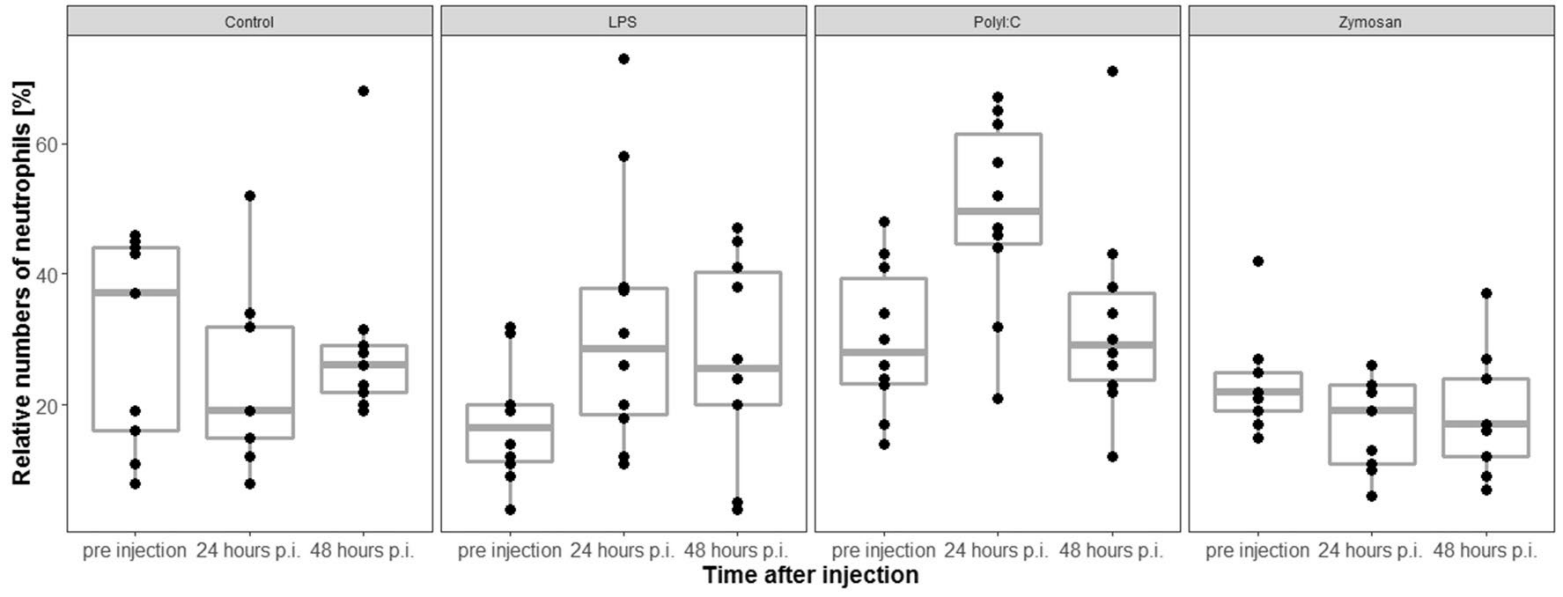
Relative numbers of neutrophils significantly increased in LPS- and PolyI:C injected individuals compared with control individuals. Relative numbers of neutrophils significantly increased in LPS- and PolyI:C injected individuals compared with zymosan injected individuals. p.i. = post injection. The figures show medians (bold line), 25–75% percentiles (box), maxima and minima (whiskers) and data points (dots).

Relative numbers of neutrophils (%) did significantly differ across treatment groups before injection of antigen/saline solution (LRT, df = 3, χ^2^ = 45.86, *p* < 0.001, n = 38, Fig. 7). Relative numbers of neutrophils tended to be lower in the LPS groups, thus in these animals there was a higher potential for an increase in neutrophil numbers compared with the other groups after injection of the antigen/saline solution.

The interaction between treatment and sampling time was significant (LRT, model with interaction vs. model without interaction: df = 3, χ^2^ = 8.44, *p* = 0.038). Because we were only interested in comparisons across treatments for the same sampling time, we restricted our post hoc analyses to comparisons among treatment at 24 h and 48 h p.i., respectively.

Relative numbers of neutrophils significantly increased in individuals treated with LPS or PolyI:C compared with individuals treated with saline solution 24 h p.i. (GLHT, LPS vs. saline solution: Estimate = 21.81, SE = 7.28, z = 3.0; *p* = 0.029; PolyI:C vs. saline solution: Estimate = 25.96, SE = 7.28, z = 3.56; *p* = 0.004). Relative numbers of neutrophils significantly increased in individuals treated with Poly I:C and LPS compared with individuals treated with zymosan where numbers decreased (GLHT, PolyI:C vs. zymosan: Estimate = 25.4, SE = 7.28; z = 3.49; *p* = 0.005; LPS vs. zymosan: Estimate = 21.25, SE = 7.28; z = 2.92; *p* = 0.036). There was no significant difference between other combinations of treatment groups (GLHT, Estimate < 14.78; *p* > 0.31, respectively).

### Relative numbers of monocytes

**Figure 8:**
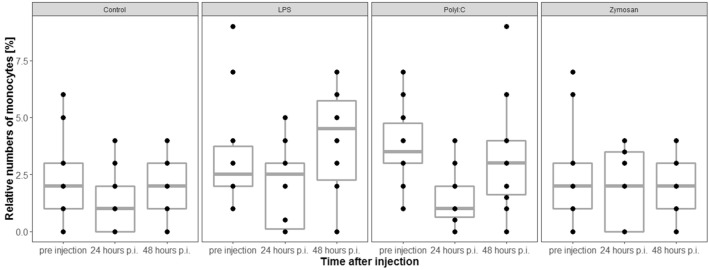
Relative numbers of monocytes were not associated with antigen type. p.i. = post injection. The figures show medians (bold line), 25–75% percentiles (box), maxima and minima (whiskers) and data points (dots).

Relative numbers of monocytes (%) did not differ across treatment groups before injection of antigen/saline solution (LRT, df = 3, χ^2^ = 4.66, *p* = 0.20, n = 38). Treatment and the interaction between sampling time and treatment did not affect the change in relative numbers of monocytes of individuals compared to before the injection (LRTs, model with interaction vs. model without interaction: df = 3, χ^2^ = 4.1, *p* = 0.25; model with treatment vs. model without treatment: df = 3, χ^2^ = 1.14, *p* = 0.77, Fig. 8).

## Discussion

This study focused on the APR in greater mouse-eared bats (*Myotis myotis*) to three antigens mimicking bacterial (LPS), viral (PolyI:C) and fungal infections (zymosan). Confirming our hypothesis, we found that the APR varied with antigen identity. Individuals challenged with LPS and PolyI:C showed a response in the measured immune markers, i.e. a shift of relative numbers from lymphocytes to neutrophils, whereas in bats challenged with zymosan, we found a decrease in relative neutrophil numbers. In the zymosan and control group, haptoglobin levels decreased compared to LPS-challenged animals. Moreover, antigen challenges in this bat species had no effect on skin temperature, total leukocyte counts and relative numbers of monocytes and did not cause changes in body mass and in the function of the constitutive innate immunity (assessed by BKA).

Haptoglobin is an acute phase protein that usually occurs at low concentrations, but production and secretion is elevated in response to acute infection and trauma^23,50^. Besides its function in the APR (reducing oxidative damage by binding hemoglobin released during hemolysis and immunomodulatory effects), haptoglobin inhibits bacterial growth^23^. Additionally, it has an important role in the anti-fungal defense of hibernating bats^42,51,52^. Haptoglobin levels may have been elevated after capture and handling in all individuals; in a previous study where *M. myotis* were housed for 5 months prior to the experiment, pre-injection levels were much lower than in our study^42^. The decrease of haptoglobin levels in the control group during the experiment may reflect the reduction of stress after habituating to the captive conditions.

### Acute phase response to LPS

The injection of LPS was associated with a change in the relative numbers of neutrophils (increase) and lymphocytes (decrease). A comparison with other studies is difficult because studies differed in experimental conditions, e.g. dosage, time of injection, and species used. Our dose was lower compared with the majority of those previously used for bats (see Table 1)^29,44,53–60^, but apparently sufficient to elicit an APR as indicated by the shift from lymphocytes to neutrophils following injection and increase in haptoglobin levels (see below). Further studies are needed to better understand whether the quality (e.g. how many and which type of components activated) and the quantity of APR in bats is dose-dependent and how this explains the intra- and interspecific differences observed. Taking into account the circadian dynamics of the immune response^61^, results from other studies may differ from ours due to differences in the time of injection. For example, individuals of a closely related species, fish-eating bat (*Myotis vivesi*)*,* were injected in the morning^46^, when bats naturally go into the dormancy phase, whereas we did during night (most active phase). Moreover, the different environmental conditions and life-history traits of species could further explain the differences in the outcome of APR to LPS injection in various bat species. Although all studied species were free-ranging, they largely differed in feeding ecology, colony size, roost conditions and geographic range, factors with known effect on immunity^34,45,62–64^. Therefore, each species may have adapted to specific challenges of pathogens, which is rather surprising since this response is well conserved among different taxa. In Table 1, we summarized the current knowledge on immune indices upon LPS challenge in bats.

**Table 1:** Comparative data of LPS challenge studies in various bat species where immunological outcomes were measured.

	Myotis myotis*	*Myotis vivesi*^46,54^	*Pipistrellus nathusii*^55^	*Carollia perspicillata*^44,59,66^	*Artibeus lituratus*^56^	*Desmodus rotundus*^60^	*Glossophaga soricina*^58^	*Molossus molossus*^29^	*Chaerephon plicatus*^57^	*Rousettus aegyptiacus*^53,67^
Geographic range	Temperate	Subtropics	Temperate	Tropics	Tropics	Tropics	Tropics	Tropics	Tropics	Subtropics—tropics
Feeding ecology	Insectivorous	Piscivorous	Insectivorous	Frugivorous	Frugivorous	Sangivorous	Nectarivorous and frugivorous	Insectivorous	Insectivorous	Frugivorous
Average body mass (g)	24	25	10	20	65	33	10	13	22	125
Duration of experiment (h)	48	11^46^; 11.5^54^	24	24^44^; 9^59^; 24^66^	24	24	10	24	8	48
Dosage (mg/kg bw)	1	1.75	2.5	2^44^; 2.7–3.3^59^; 3.1^66^	2	5	2.84	4.5	2.27	2 and 4^adults;^^53,67^, 2^juveniles,^^53^
Sex	Females	Females and males	Females^pre-migration^; females and males^migration^	Females and males^44,59^; males^66^	Females and males	Females	Females and males	Females and males	Females and males	Females and males^53^; males^67^
Time of injection	Evening	Morning	Evening	No information available^44^; morning^59^; evening^66^	No information available	Night	Morning	Evening	Morning	Morning and evening
Samples size/group	9	7 (repeated)	9^pre-migration^; 14^migration^	20^44^, 31 (repeated)^59^; 9 (repeated)^66^	10–11	6–14 (repeated)	6–7	6–7 (temperature, bw, leukocytes)/16–17 (bw, leukocytes)	21–22	10-11^adults,^^53,67^; 5^juveniles,^^53^
Route of injection	Subcutaneous	Subcutaneous	Subcutaneous	Subcutaneous	Subcutaneous	Subcutaneous	Subcutaneous	Subcutaneous	Intraperitoneal	Subcutaneous
Temperature	ns	+	NM	NM^44^; + ^59^; ns^66^	+	NM	NM	ns	NM	+ ^53^; NM^67^
Body mass	ns	–	ns^pre-migration^; -^migration^	–	–	–	–	–	ns	− ^53^; NM^67^
Total leukocyte counts	ns	NM	NM	+ ^44^; ns^59^; ns^66^	ns	+	NM	ns	+	ns^53^; NM^67^
Lymphocytes	–	NM	+ ^pre-migration^; ns^migration^	NM^44^; − ^59^; − ^66^	NM	–	NM	NM	n.s	-^adults^, ns^juveniles^^53^ ; NM^67^
Neutrophils	+	NM	-^pre-migration^; ns^migration^	NM^44^, + ^59^; − ^66^	NM	+	NM	NM	+	+ ^adults^, ns^juveniles^^53^; NM^67^
Monocytes	ns	NM	NM	NM	NM	NM	NM	NM	ns	ns^53^ ; NM^67^
Haptoglobin	+	NM	ns^pre-migration^; + ^migration^	NM	NM	NM	NM	NM	NM	+
Lysozyme	NM	NM	NM	NM	NM	NM	NM	NM	NM	+
BKA	ns	NM	NM	NM	NM	NM	NM	NM	NM	NM

*Current study, ns = non-significant, + = significant increase, − = significant decrease, NM = not measured, bw = body weight.

A simulated bacterial infection with LPS elicited an immune response in several vertebrate species (e.g. house sparrow^32^; great tit (*Parus major*)^65^; fish-eating bat^54^; Seba’s short-tailed fruit bat (*Carollia perspicillata*)^44,66^; Egyptian fruit bat (*Rousettus aegyptiacus*)^53,67^*;* African mole rats (*Cryptomys hottentotus pretoriae*)^68^; mice (*Mus musculus*) and humans^27^) although the number of immune and physiological markers measured varies between studies. Similar to Pallas’s mastiff bats (*Molossus molossus*)^29^, skin temperature was not affected by LPS treatment in greater mouse-eared bats. The lack of a fever response to LPS challenge in some bat species is contrasting with other vertebrate taxa and could be associated with the apparently unique absence of the PYHIN gene family in bats^69^. Translation of genes of the PYHIN family results in non-bat taxa in immune sensors that activate the inflammasome and/or interferon pathways^69^. Thus, some bat species may neither sense intracellular foreign DNA particles nor mount an (innate) immune response in the same way as other vertebrates. However, LPS challenged Egyptian^53^, short-tailed^59^ and great fruit-eating bats (*Artibeus lituratus*)^56^ and fish-eating bats^46^ showed a febrile response. Additionally, fever was documented in great fruit-eating bats during mimicked viral infection^43^ (see below), while febrile cytokine IL-6 was significantly expressed in hibernating little brown bats (*Myotis lucifugus*) infected with *P. destructans*^70^ which might lead to fever^71^. Fever can lead to an increase in the metabolic rate and thus may result in the loss of body mass^46^. The loss of body mass can also be related to elevated levels of pro-inflammatory cytokines that trigger protein and energy mobilization to allow increased body temperature, thus reducing fat stores and muscle tissue^72,73^.

Yet, fever and body mass loss in response to LPS injection are not stringently correlated. For example, in house sparrows the immune response to LPS injection included nocturnal hyperthermia, but no change in body mass^36^. In Pallas’s mastiff bats, body mass decreased upon LPS challenge, but body temperature was not affected^29^. In Pallas’s long-tongued bats (*Glossophaga soricina*), house sparrows and white-crowned sparrows (*Zonotrichia leucophrys gambelii*) body mass decreased upon LPS challenge, but temperature was not assessed^32,35,58^. In the wrinkled-lipped bat (*Chaerephon plicatus*), LPS injection was not associated with body mass loss, although this finding may be related to the relatively short study period of 8 hours^57^ compared with the other studies (11–24 h, see Table 1). In summary, the depletion of somatic resources upon mimicked bacterial infection may be context-dependent (environmental factors and other life-history traits) and not consistent among species.

A loss in body mass can be related to other factors besides fever. Firstly, antigen-induced sickness behaviour can lower the appetite of individuals (anorexia), resulting in reduced food consumption^53^. Secondly, immune challenges are metabolically costly^28^ and thus a decrease in body mass may reflect the mobilization of energy reserves to fuel the immune response, e.g. as shown in leukocytosis. Indeed, leukocyte counts increased after injection with LPS in great tits^65^, short-tailed fruit bats^44^, common vampire bats (*Desmodus rotundus*)^60^ and wrinkle-lipped free-tailed bats^57^. However, in our study on greater mouse-eared bats, in Egyptian fruit bats, Pallas’s mastiff bats and great fruit-eating bats, no evidence for a cellular immune reaction reflected by total leukocyte counts was found in response to the LPS challenge^29,53,56^.

Like in Egyptian fruit bats, short-tailed fruit bats and common vampire bats, we observed an increase in the neutrophil to lymphocyte ratio in greater mouse-eared bats following LPS challenge^53,59,60,66^. In wrinkle-lipped free-tailed bats, LPS injected animals also showed an increase in neutrophils, but no significant change in lymphocyte or monocyte numbers^57^.

Similarly to previous bat studies measuring haptoglobin levels after LPS injection^53,55,67^, we also observed a rise in this acute phase protein in greater mouse-eared bats. Haptoglobin can be considered a major acute phase protein in bats, which is due to its relatively low energetic cost it is mainly activated during periods with high energy demand such as migration^55^ or hibernation^42^. In contrast to a study on red crossbills (*Loxia curvirostra*) who found significantly elevated levels of BKA in LPS injected birds^74^, the BKA in greater mouse-eared bats was not associated with this immune challenge. Overall, the reaction to an LPS challenge seems to vary interspecifically even among species of the same taxon.

### Acute phase response to PolyI:C

In contrast to our prediction, the challenge of PolyI:C caused an increase in the relative numbers of neutrophils and decrease in the relative number of lymphocytes in our study. We predicted no effect since it has been shown that bats have a constitutive expression of the interferon type I (IFN α and β) with antiviral activity^17^. Neutrophils are part of the first line innate immune defence and are the main phagocytic leukocyte type^21^. These phagocytes kill pathogens (including viruses) either by engulfing and subsequently digesting them in a phagosom, by secretion of different antimicrobial substances or with neutrophil extracellular traps^75^. Neutrophils proliferate after infection, stress and inflammation^21,76^. Consistent with the study on house sparrows^36^, we did not find an effect of PolyI:C injection on body mass, skin temperature and haptoglobin. However, in a recent study, great fruit-eating bats were challenged by a lower dosage of PolyI:C^43^. Animals injected with this antigen not only increased their resting metabolic rate 6 h post injection, but also lost more body weight compared to the control animals and showed an increase in body temperature^43^. Moreover, there were no differences between treatment groups at 24 h post injection, indicating a quick response towards viral antigens in this bat species. Since we assessed most APR markers 24 h and 48 h post injection, we might have missed such a response, an aspect which needs to be studied in the future.

### Acute phase response to zymosan

The injection of zymosan resulted in no immune response in our study which is consistent with the study on house sparrows, were no APR towards zymosan was found^36^; however this outcome is strikingly different from the results of a recent study on hibernating greater mouse-eared bats^42^.When injected with the similar dosage of zymosan, hibernating greater mouse-eared bats increased the circulating levels of haptoglobin compared with control animals^42^. This result is not only surprising since immune functions are usually down regulated during hibernation^77^, but also due to the opposing results observed between active and torpid individuals.

## Conclusions

Despite our predictions to find no APR to viral, but to fungal and bacterial challenges, we found a reaction to the viral and the bacterial antigens. However, it is noteworthy that other proteins, e.g. from other fungi or gram-positive bacteria, might elicit an APR differently than those used in this study. Additionally, the route of administration of the antigen may play a role in the initiation of an APR. Whereas we injected the antigens subcutaneously, natural infections usually occur via other routes (e.g. oro-fecal, respiratory).

Remarkably, none of the antigens elicited a fever response. Thus, we confirm our hypothesis and add to the observation that bats, depending on their ecological niche, may have adapted to not react with an APR to certain infections and/or respond with adjusted defense mechanisms^42,52,55^. Possibly, the benefits of fighting certain pathogens do not outweigh the costs of self-defense (energetic and pathological). Consequently, it may not be adaptive for bats to raise an immune response to all pathogens in a similar way. Future studies could investigate the molecular pathways in more detail using new tools^51,70,78–80^ both in vitro and in vivo. For example, the effect of antigen challenges on the expression and secretion of pro- and anti-inflammatory cytokines should be studied to better understand the underlying molecular mechanisms of the immune defense in bats, especially towards different pathogen groups.

## Methods

### Experimental setup

In August 2015, we captured 38 non-lactating adult female greater mouse-eared bats (*Myotis myotis*) at the Orlova Chuka cave, using harp traps, in cooperation with the bat research station in Tabachka, Bulgaria, administered by Dr. H. Goerlitz (Max Planck Institute for Ornithology, Seewiesen, Germany). We chose the greater mouse-eared bat as model species because its size (20–30 g) allowed us to repeatedly take sufficiently large blood samples. Work was granted under the permit 639/28.05.2015 issued by the Directorate of the Rusenski Lom Nature Park (Direktor Tsonka Hristova), Bulgaria. We housed bats individually in plastic boxes where we fed them daily with mealworms and provided water ad libitum. The experiment started after the bats had been allowed to habituate for about 36 h. To trigger APRs, we used three pathogen-associated molecular patterns (PAMPs) to mimic bacterial, fungal and viral infections: LPS (an endotoxin of gram-negative bacteria cell walls), zymosan (a fungal glucan) and polyinosinic:polycytidylic acid (PolyI:C, a synthetic double-stranded RNA). We assigned individuals randomly to the four experimental groups (LPS: n = 9, zymosan: n = 10, PolyI:C: n = 9 and saline solution as a control: n = 10). We compared indices of the APR (number and type of leukocytes, haptoglobin concentration, fever, change in body mass) and the overall function of the constitutive innate immunity via bacterial killing activity of plasma between 4 groups of bats either injected with LPS, PolyI:C, zymosan or saline solution as a control (see below). Most of the previous studies measured the APR within 24 h post-injection period^29,44^, so in order to elucidate potential long-term responses in bats, we followed the animals for 48 h^53^.

We equipped bats with temperature-sensitive radiotransmitters (LB-2XT, Holohil Systems Ltd., Carp, ON, Canada) which emit signal pulse rates as a function of the animal’s skin temperature. We used an Australis 26 k scanning receiver, an omni-directional antenna, and a Titley Scientific RF data logger to automatically record the transmitter pulse rates which correspond to skin temperatures of individual bats throughout the duration of the experiment. The skin temperature is a reliable proxy of body temperature in torpid and active bats^53,81^. To attach the radio transmitters, we first removed a tuft of fur from the interscapular region and then glued the radio transmitter with medical skin glue (Manfred Sauer GmbH, Germany) onto the furless skin.

Before applying the challenge, we weighed bats and collected a blood sample of about 70 µl using heparinised capillaries from each individual. We used 3 µl of blood to produce a smear for total and differential leukocyte counts (see below). We centrifuged the remaining blood to separate plasma from cellular components and froze both fractions in a dry shipper at − 196 °C until being transported to Germany, where samples were stored at − 80 °C until further analysis. We injected the different solutions subcutaneously using a sterile disposable syringe (no. 00531, Carl Roth GmbH, Karlsruhe, Germany) between 8 pm and midnight. Animals from the LPS group received 30 µl of LPS (*Escherichia coli*, no. L2630, Sigma-Aldrich, Munich, Germany) in saline solution resulting in a concentration of 1 mg/kg body mass. The zymosan group received 21 µl of zymosan (no. 58856-93-2, Invivogen, Tolouse, France) in saline solution, in a dosage of 0.7 mg/kg body mass, the PolyI:C group 190 µl of PolyI:C (no. 31852-29-6, Invivogen, Tolouse, France) in saline solution, in a dosage of 25 mg/kg body mass and the control group 80 µl of sterile saline solution. These dosages were selected because they have been successfully used before in a similarly sized bird species, the house sparrow (*Passer domesticus*)^36^.

We weighed the bats and collected from each a second and third blood sample 24 and 48 h p.i. (procedure of preparing and storing samples as before). After 48 h, we removed radio transmitters and after confirming a good health status we released all bats at the site of capture.

### Bacterial killing activity (BKA)

The BKA is a constitutive innate marker of the immune system and measures humoral and cellular components in function of the sample used. While using whole blood is possible to quantify the overall constitutive innate immunity of an individual^82^, with serum or plasma samples only the humoral part is measured^83,84^. We assessed the bacterial killing activity (BKA) of the plasma against *E. coli *in vitro following the method of Schneeberger et al.^62^. Plasma samples were diluted 1:50 in sterile PBS and we added 10 µl of a suspension of living *E. coli* (ATCC #8739) to each diluted sample (140 µl). The bacterial suspension was adjusted to a concentration of ~ 200 colonies per 50 µl plasma-bacteria mixture. The mixtures were then incubated for 30 min at 37 °C. After incubation, 50 µl aliquot of the vortexed mixture was spread onto Tryptic Soy Agar plates in duplicate, followed by overnight incubation at 37 °C. To obtain the initial number of bacteria that we had before starting to interact with the plasma, we diluted 140 µl PBS with bacterial suspension and plated in similar ways. On the following day, the colony-forming units were counted and the bacterial killing activity was defined as percent of the killed bacteria, which was calculated as 1-(average of viable bacteria after incubation / the initial number of bacteria)^62^.

### Haptoglobin

Haptoglobin is an acute phase protein that usually occurs at low concentrations, but production and secretion is increased in response to acute infection and trauma, including in bats^23,42,50,53,67^. As an acute phase protein, haptoglobin reduces oxidative damage by binding hemoglobin released during hemolysis, has immunomodulatory effects and inhibits bacterial growth. To measure the concentration of haptoglobin, we followed the standard procedure of the commercial kit "PHASE" ™ Haptoglobin Assay (Cat. No. TP-801, Tridelta, Maynooth, Ireland) using a colorimetric assay^42,53,55^.

### Total and differential leukocyte counts

Total and differential leukocyte (white blood cell) counts are considered to be indicators of health status, current infectious processes, stress and trauma^76,85^. Neutrophils are the first line of the innate defense against pathogens and parasites; their number increases especially during bacterial and fungal infections. Lymphocytes are the cellular effector of the adaptive immune system, their number increases mainly in viral infections. Monocytes, eosinophils and basophils are minor leukocyte types, with specific functions as phagocytic cells, antiparasitic defense and allergy.

We stained blood smears with May-Gruenwald’s solution (no T863.2, Carl Roth GmbH, Karlsruhe, Germany) and Giemsa (no. T862.1, Carl Roth GmbH, Karlsruhe, Germany). Blood smears were analyzed with a microscope under oil immersion at a 100 × magnification. Blood smears were analyzed blindly and conducted by the same person. We estimated the total leukocyte counts manually by the mean number of white blood cells per visual field using the total count of immune cells in 10 fields^62^. The number of leukocytes obtained by this method corelates with numbers obtained by conventional methods^62^.

For the differential leukocyte counts, we counted 100 leukocytes and identified the different types of immune cells by size, color, shape and cytoplasmic contents. From this, we calculated relative numbers (%) of lymphocytes, neutrophils, eosinophils, monocytes and basophils. Further on, we exclusively consider lymphocytes, neutrophils and monocytes in the comparison between treatments because eosinophils and basophils occurred at very low concentrations in the blood (mean of 3% and 7%, respectively).

### Ethical approval

All experimental procedures described in the materials and methods section were approved by the *Internal Committee for Ethics and Animal Welfare* of the Leibniz Institute for Zoo and Wildlife Research (permit #2015-03-05). All experiments were carried out in accordance with the approved guidelines of the IZW and comply with the laws of Bulgaria. The reporting in the study follows the recommendations in the ARRIVE guidelines.

### Statistics

We used the statistical software R version 3.5.1 for all statistical analyses^86^. We conducted two-tailed tests and set the level of significance to α = 0.05. We pooled the data of skin temperature to 4-h intervals for analyses. We used a linear mixed-effects model using the package “lme4”^87^ with temperature as response variable. We included treatment (control, LPS, PolyI:C, zymosan), time since injection (4-h intervals) and their interaction as predictor variables. Additionally, we included the identity of the bats as a random variable to control for inter-individual differences. We analyzed the model using an ANOVA (Type I).

For all other response variables (body mass, BKA, haptoglobin, total leukocyte counts, relative number of monocytes, neutrophils and lymphocytes), we analyzed the data also using the package “lme4” using linear mixed-effects models. The response variable was the difference in the measured value at 24 or 48 h, respectively, compared with the value before injection. For the response variables total leukocyte counts and haptoglobin (mg/ml), we transformed the response variable using Box-Cox power transformation to improve model assumptions (normal distribution of residuals, normal distribution of random intercepts and homogeneity of variance) using the function “powerTransform” of the R package “car”^88^. The predictor variables were time of sampling (24 and 48 h p.i.), treatment and their interaction. Additionally, we included the identity of the bats as a random variable to control for inter-individual differences. In case that treatment or the interaction between treatment and sampling time did not have a significant effect on the response variable, we removed it from the model to improve model interpretation. In case that there was a significant effect of treatment or the interaction between treatment and sampling time we applied a Tukey’s post-hoc test to compare factor levels with p-value adjustment in the R package “multcomp”^89^.

Before these analyses, we tested if measurements in individuals differed before injection across the four treatment groups using a LRT of two linear mixed effects model assuming a gaussian (body mass, haptoglobin) or poisson distribution (BKA, total leukocyte counts, relative number of monocytes, neutrophils and lymphocytes), respectively, whereas we compared a null model with the model including treatment as fixed effect, respectively. We included individual identity as a random factor only in the BKA models because this was the only measurement where we had two data points for each sample.

## Data Availability

The datasets generated during the current study are available from the corresponding author on reasonable request.
