# Author Correction: Differences in acute phase response to bacterial, fungal and viral antigens in greater mouse-eared bats (*Myotis myotis*)

**DOI:** 10.1038/s41598-022-25685-2

**Published:** 2022-12-07

**Authors:** Anne Seltmann, Sara A. Troxell, Julia Schad, Marcus Fritze, Liam D. Bailey, Christian C. Voigt, Gábor Á. Czirják

**Affiliations:** 1 Department of Evolutionary Ecology, Leibniz Institute for Zoo and Wildlife Research, Alfred-Kowalke-Str. 17, 10315 Berlin, Germany; 2 Institute of Biology, Freie Universität Berlin, Takustr. 3, 14195 Berlin, Germany; 3 Department of Evolutionary Genetics, Leibniz Institute for Zoo and Wildlife Research, Alfred-Kowalke-Str. 17, 10315 Berlin, Germany; 4 Department of Wildlife Diseases, Leibniz Institute for Zoo and Wildlife Research, Alfred-Kowalke-Str. 17, 10315 Berlin, Germany; 5 Present Address: Department 3: Public Relations and Environmental Education, National Park Administration Saxon Switzerland, An der Elbe 4, 01814 Bad Schandau, Germany; 6 Present Address: Applied Zoology and Nature Conservation, University of Greifswald, Loitzer Str. 26, 17489 Greifswald, Germany

Correction to: *Scientific Reports* 10.1038/s41598-022-18240-6, published online 10 September 2022

The original version of this Article contained an error in the Acknowledgements section, where

“We are very grateful to the Directorate of the Rusenski Lom Nature Park (Director Tsonka Hristova) for cooperation and support. We thank the responsible Bulgarian authorities (MOEW-Sofia and RIOSV-Ruse) for granting us permission to conduct this research. We would like to thank Stefan Greif for his support in capturing bats in Bulgaria and Katja Pohle for her help in the laboratory analyses. This work was supported by institutional funds of the Leibniz Institute for Zoo and Wildlife Research and the German Research Foundation (DFG Priority Programme 1596). Funding for the research station in Tabachka was provided by the DFG to Holger R. Goerlitz (Emmy Noether Program GO2091/2-1). Open Access funding enabled and organized by Projekt DEAL and was funded by the Deutsche Forschungsgemeinschaft (German Research Foundation–Projektnummer 491292795).”

now reads:

“We are very grateful to the Directorate of the Rusenski Lom Nature Park (Director Tsonka Hristova) for cooperation and support. We thank the responsible Bulgarian authorities (MOEW-Sofia and RIOSV-Ruse) for granting us permission to conduct this research. We would like to thank Stefan Greif for his support in capturing bats in Bulgaria and Katja Pohle for her help in the laboratory analyses. This work was supported by institutional funds of the Leibniz Institute for Zoo and Wildlife Research and the German Research Foundation (DFG Priority Programme 1596). Funding for the research station in Tabachka was provided by the DFG to Holger R. Goerlitz (Emmy Noether Program GO2091/2-1).”

Additionally, the Funding section in the original version of this Article was incomplete.

“Open Access funding enabled and organized by Projekt DEAL.”

now reads:

“Open Access funding enabled and organized by Projekt DEAL. The publication of this article was funded by the Deutsche Forschungsgemeinschaft (DFG, German Research Foundation) - project number 491292795.”

The original Article has been corrected.

